# Facilitators and Barriers to Over-the-Counter Hearing Aid Use in People With Dementia: Semistructured Interview Study

**DOI:** 10.2196/83857

**Published:** 2026-04-01

**Authors:** Dana P Urbanski, Alexander M Hungs, Peggy B Nelson, Joseph E Gaugler

**Affiliations:** 1 Department of Speech, Language and Hearing Sciences College of Arts and Sciences Indiana University Bloomington Bloomington, IN United States; 2 Regenstrief Institute Indiana University Center for Aging Research Indianapolis, IN United States; 3 University of Minnesota School of Public Health Center for Healthy Aging and Innovation Minneapolis, MN United States; 4 Department of Speech-Language-Hearing Sciences College of Liberal Arts University of Minnesota Minneapolis, MN United States; 5 University of Minnesota Center for Applied and Translational Sensory Science Minneapolis, MN United States; 6 Division of Health Policy and Management School of Public Health University of Minnesota Minneapolis, MN United States; 7 Center for Healthy Aging and Innovation School of Public Health University of Minnesota Minneapolis, MN United States

**Keywords:** over-the-counter hearing aids, dementia, hearing loss, older adults, caregiver, caregiving, assistive technology

## Abstract

**Background:**

Over-the-counter (OTC) hearing aids were introduced to improve the affordability and accessibility of hearing health care for adults with perceived mild-to-moderate hearing loss. While these devices have demonstrated effectiveness in cognitively healthy older adults—particularly in the domains of audibility, self-reported hearing ability, and speech recognition in quiet—their use and outcomes in people with dementia remain underexplored. This issue warrants further attention, as people with dementia often experience co-occurring hearing loss and may rely on OTC hearing aids to overcome cost and access barriers to prescription amplification. However, given the cognitive and functional challenges of dementia, it is unclear whether and how OTC hearing aids can support the hearing care needs of these individuals.

**Objective:**

To explore interest-holder perspectives on the feasibility and acceptability of OTC hearing aids for community-dwelling older adults with dementia, identifying key facilitators and barriers that influence their use in this population.

**Methods:**

Semistructured interviews were conducted with 45 participants across three interest-holder groups (15 per group): (1) community-dwelling older adults with dementia and hearing loss, (2) family caregivers of community-dwelling older adults with dementia and hearing loss, and (3) geriatric direct care professionals. Interviews were conducted and recorded via secure Zoom (Zoom Communications) videoconferencing, then transcribed and analyzed using thematic analysis.

**Results:**

Participants endorsed several facilitators and barriers to OTC hearing aid use in people with dementia. Facilitators included increased accessibility, perceived affordability and value, and enhanced autonomy and control. Barriers included mistrust of OTC hearing aids, difficulty assessing candidacy due to unreliable self- and proxy reports of hearing status, caregiver uncertainty regarding device programming and adjustment, challenges evaluating device effectiveness, and concerns about caregiver burden and burnout from long-term device management.

**Conclusions:**

OTC hearing aids offer meaningful advantages for people with dementia and their family caregivers. However, significant barriers must be addressed to ensure their feasibility and acceptability for this population. Future research should further examine and quantify these barriers to inform the development of tailored devices, services, and delivery models that promote successful OTC hearing aid use in people with dementia and their family caregivers.

## Introduction

### Background

Newly introduced over-the-counter (OTC) hearing aids have shown effectiveness among cognitively healthy older adults with mild-to-moderate hearing loss, particularly in the domains of audibility, self-reported hearing ability, and speech recognition in quiet [1-5]. However, there is limited evidence for their use in people with dementia. In this manuscript, we explore the perspectives of dementia-care interest-holders [6] on the benefits, challenges, and opportunities of OTC hearing aid use in community-dwelling people with dementia.

### OTC Hearing Aids: Reducing Cost and Improving Accessibility

Among American older adults, age-related hearing loss affects about two-thirds of people older than 70 years and more than 80% of those aged 80 years and older [7,8]. For most of these individuals, hearing aids are the most effective therapy for age-related hearing loss [9], offering well-documented health benefits [10-13]. However, hearing aid adoption is low among older Americans with hearing loss—typically ranging from 30% to 40% [8,14,15]. To address this problem, in October 2022, the United States Food and Drug Administration (FDA) introduced regulations for OTC hearing aids, creating a new category of hearing aids available directly to consumers without a prescription. Previously, all hearing aids required a professional evaluation and fitting by a licensed hearing care professional. Today, OTC hearing aids—now widely available in stores and web-based retailers—allow adults with self-perceived mild-to-moderate hearing loss to purchase and fit hearing aids of their choosing. This change is intended to make hearing aids more affordable and accessible by removing the added expense and travel associated with seeing a licensed professional. Proponents of the new regulations anticipate that more affordable, widely available OTC hearing aids will substantially lower cost and access barriers to amplification, thereby facilitating greater hearing aid uptake among approximately 15 million Americans living with untreated hearing loss [8].

The reasons for low hearing aid adoption are multifactorial and include barriers other than cost and access, such as stigma, low readiness to address hearing loss, and poor hearing aid self-efficacy, among other factors [16-19]. Nevertheless, cost and access are formidable barriers to prescription hearing aids for many older adults [20,21]. First, conventional prescription hearing aids are expensive and not covered by Medicare. On average, a pair of prescription devices costs US $3600 [22]. This price may be cost-prohibitive for many older Americans with low or fixed incomes. Indeed, a recent analysis found that an out-of-pocket cost of US $2500 for hearing aids is a catastrophic, unaffordable expense for upwards of three-quarters of American adults with hearing loss [21]. Moreover, prescription hearing aids are geographically inaccessible to many Americans. Recent data indicate that audiologists are unevenly and inequitably distributed across the United States, clustering in metropolitan areas with low rates of self-reported hearing difficulty to the disservice of rural areas with greater self-reported hearing difficulty [23]. In short, those in rural or underserved communities may lack access to prescription hearing aids, irrespective of cost or insurance coverage. Together, the poor affordability and inequitable availability of prescription hearing health care necessitate an alternative approach. For many older Americans, OTC hearing aids offer this much-needed alternative.

### OTC Hearing Aids: Considering the Needs of People With Dementia

While OTC hearing aids address cost and access, they also carry broader implications for older adults with hearing loss. OTC hearing aids introduce a new “do-it-yourself” service delivery model in which older adults assume responsibility for selecting, fitting, using, managing, and troubleshooting hearing aids. To date, a small but growing body of research shows that well-selected, generally healthy older adults with intact cognition can successfully self-program, use, and manage OTC hearing aids [1-5]. However, OTC hearing aids are not tested or designed for people with dementia, who may lack the cognitive ability to understand and use these devices. Although traditional prescription hearing care can also present challenges for people living with dementia, the lack of clinician involvement in OTC hearing aids introduces potential challenges that remain unexamined. This gap in the evidence for OTC hearing aids warrants further consideration. Older people with co-occurring dementia and hearing loss are a critical subpopulation of people with untreated hearing loss who—like the general older adult population—will rely on OTC hearing aids to overcome existing cost and access barriers to prescription hearing aids. If people with dementia are to benefit from OTC hearing aids, it is essential to explore their needs, perspectives, and capabilities for using these devices.

### OTC Hearing Aids for People With Dementia: Potential Benefits, Challenges, and Opportunities

Like age-related hearing loss, dementia is prevalent in older adults. Approximately 10% of American older adults aged 65 years and older live with dementia, increasing with age to 20% of those aged 80 years and older and 40% for those older than 90 years [24-26]. Many individuals with dementia also experience co-occurring hearing loss. Among those older than 70 years with dementia, 79% have clinically significant hearing loss, rising to 94% of those aged 85 years and older [27]. For those with both conditions, untreated hearing loss can compound the effects of dementia on communication, exacerbating dementia-related behavioral and emotional changes such as depression, agitation, apathy, and social withdrawal [28-30]. Importantly, hearing aids may relieve the compounding effects of hearing loss on dementia. In people with dementia, hearing aid use is associated with reduced depressive, neuropsychiatric, and other problematic behavioral symptoms [28,31-33]. These health benefits broadly mirror the social and emotional health benefits observed for hearing aid use in the general older adult population. However, fewer than one-third of people with co-occurring dementia and hearing loss use hearing aids [27,34]. In short—like the general older adult population—people with dementia benefit from hearing aids but demonstrate low adoption rates while facing high cost and access barriers to prescription hearing aids. Although people with dementia may face additional barriers to hearing aid adoption, cost and access are important barriers for people with and without dementia. It is thus essential to explore whether and how OTC hearing aids can provide inclusive access to affordable hearing health care for people with dementia.

It remains unclear whether OTC hearing aids are feasible and acceptable for community-dwelling people with dementia and their family caregivers. Without robust evidence on this topic, one can draw parallels between OTC hearing aids and the broader literature on everyday and at-home assistive technology use in older people with dementia. Here, studies consistently find that older adults with cognitive impairment, including dementia, both demonstrate and self-report greater difficulty understanding and managing a variety of everyday technologies compared with their peers without cognitive impairment [35-37]. In quantitative studies, these findings are statistically significant and show large effect sizes [35,37,38]. In qualitative studies, older adults with cognitive impairment report an array of overlapping, compounding challenges for at-home technology use, including memory deficits, difficulty attending to multiple processes, inability to follow sequential instructions, confusion interpreting technology-generated messages, low self-efficacy for technology use, and sensitivity to technology-related stress, among others [36,39,40]. Some people with cognitive impairment even forget what a given technology is intended to do or how it is meant to function [36]. Importantly, studies have found that manufacturer instruction manuals are typically unhelpful for older adults with cognitive impairment, including dementia, who may express optimism in their ability to follow written instructions but often fail to implement them correctly [36].

Considering these challenges, people with dementia will likely need family or other caregiver support to use OTC hearing aids. This is not unexpected, as people with dementia typically need help performing some or all instrumental activities of daily living (IADL), such as housekeeping, preparing food, taking medication, using transportation, shopping, managing finances, and using the telephone [41]. Similarly, people with dementia usually require some level of caregiver assistance for using traditional prescription hearing aids [42,43]. Yet OTC hearing aids have been developed for independent use, without explicit consideration of caregiver roles. In the absence of ongoing professional hearing services and counseling, it is unclear if OTC hearing aids are designed and delivered in ways that facilitate effective family caregiver involvement. It is further unknown if family caregivers are open to and comfortable assuming responsibilities for OTC hearing aid programming, use, care, and maintenance. Research is needed that explores the needs, perspectives, and capabilities of community-dwelling people with dementia and their family caregivers for using OTC hearing aids. This knowledge can inform the development of tailored devices, services, and supports that promote successful OTC hearing aid use in people with dementia and their family caregivers, thus ensuring that people with dementia can benefit from lower-cost, more accessible OTC hearing aids.

### Study Objectives

This study explores perspectives on the feasibility and acceptability of OTC hearing aids as a pathway to hearing health care in community-dwelling older people with dementia. We conducted individual semistructured interviews with participants from 3 dementia-care interest-holder groups, including community-dwelling people with dementia and hearing difficulty, family caregivers of people with dementia and hearing difficulty, and geriatric direct care professionals with expertise in the care needs of older people with dementia and hearing difficulty. Using thematic analysis of the interview data, we aimed to identify (1) facilitators or advantages of OTC hearing aid use for community-dwelling older adults with dementia, and (2) barriers or disadvantages of OTC hearing aid use in these individuals. Study reporting follows the Consolidated Criteria for Reporting Qualitative Research (COREQ) guidelines.

## Methods

### Participant Groups and Eligibility Criteria

Participants were recruited to represent key dementia-care interest-holders directly affected by, or with knowledge pertaining to, OTC hearing aid use in community-dwelling people with dementia. Interest-holders represented three groups (15 each): (1) community-dwelling older adults with self- or caregiver-reported early to midstage dementia and self-perceived mild-to-moderate hearing loss, (2) family caregivers of community-dwelling older people with caregiver-reported early to midstage dementia and caregiver-reported mild-to-moderate hearing difficulty, and (3) geriatric care professionals who provide direct health care services to community-dwelling older adults with co-occurring dementia and hearing loss. Participants were recruited through a research registry of geriatric care professionals and family caregivers of people living with dementia, as well as via web-based advertising and word-of-mouth referrals.

To be eligible for inclusion, community-dwelling older adults with dementia met the following criteria: (1) aged 65 years or older, (2) non–nursing home resident, (3) self- or caregiver-reported diagnosis of early to midstage Alzheimer disease or related dementia, (4) education-adjusted Telephone Interview for Cognitive Status–modified (TICS-m) score of ≤27 out of 50 points consistent with the presence of dementia [44,45], (5) self-perceived mild-to-moderate hearing loss, and (6) ability to communicate in English and follow study procedures. We relied on self-perceived mild-to-moderate hearing loss rather than clinical audiometric test results to align with the FDA’s printed candidacy guidelines for OTC hearing aids. Participants were not required to provide audiometric test results and could have any or no prior or current hearing aid experience.

Family caregivers were recruited separately from older persons with dementia (ie, caregivers and individuals with dementia were not enrolled as care dyads) and met the following inclusion criteria: (1) aged 18 years or older, (2) self-identified provider of informal or unpaid health care assistance to a family member who is a community-dwelling older person aged 65 years or older with co-occurring early to midstage Alzheimer disease or related dementia and mild-to-moderate hearing difficulty, (3) self-reported negative history of cognitive impairment or dementia, and (4) able to communicate in English and follow study procedures. Family caregivers could have any or no experience using hearing aids and assisting others in using hearing aids. To ensure study feasibility and accessibility, persons with dementia and family caregivers were not required to supply medical records confirming the dementia or hearing loss status of the person with dementia.

Geriatric direct care professionals were required to be at least 18 years old, able to communicate in English, and providers of direct health care services for community-dwelling older adults, including those with co-occurring dementia and hearing loss. They could be from any profession or geriatric care specialty outside of audiology, otolaryngology, and speech-language pathology. No specific hands-on hearing aid experience or knowledge was required.

### Ethical Considerations

This study was approved by the University of Minnesota Institutional Review Board (STUDY00016265). Prior to enrollment, all participants gave informed consent or assent in accordance with institutional review board requirements. Individuals with dementia gave their verbal assent for participation, while written informed consent was obtained from a legally authorized representative. Family caregivers and geriatric direct care professionals provided written informed consent. Participant data were deidentified and stored on secure, password-protected servers to protect privacy and confidentiality. All participants were compensated for their time and contributions.

### Descriptive Measures

We collected sociodemographic information from all participants. Additionally, several screening and descriptive measures were administered to characterize participants with dementia, including (1) TICS-m [45], (2) Hearing Handicap Inventory for the Elderly (HHIE) [46], and (3) Lawton-Brody IADL Scale (Lawton and Brody [47]).

We used the TICS-m as a quantitative measure of cognitive function [45]. The TICS-m is a widely used telephone-administered screening test of cognitive functioning designed for use when in-person assessment is impractical or infeasible. The test consists of 11 items (50 points total) covering several cognitive domains, including orientation to time and place, receptive and expressive language ability, verbal memory, calculation, and verbal abstraction. Scores have shown good sensitivity for detecting dementia in older adults when using a cutoff of ≤27 points for education-adjusted TICS-m scores [44]. In this study, TICS-m scores were adjusted for years of education using the corrections in Knopman et al [44] and required to meet the ≤ 27 cutoff, providing additional evidence for participants’ cognitive function beyond their self- or caregiver-reported dementia status.

As mentioned previously, we relied on self-perceived hearing difficulty, consistent with FDA candidacy guidelines for OTC hearing aids. To provide additional descriptive information regarding the hearing abilities of participants with dementia, we administered the HHIE [46]. The HHIE is a validated self-assessment of hearing impairment consisting of 25 questions that measure situational and emotional effects of hearing loss in older adults. Each item presents a potential everyday impact of hearing loss, which the respondent rates as “yes,” “sometimes,” or “no.” Each “yes” response is worth 4 points, “sometimes” is 2 points, and “no” is 0 points, giving a maximum possible score of 100 points for the highest level of perceived hearing difficulty. The results give a useful measure of subjective hearing difficulty; however, it should be noted that the HHIE is not specifically validated for use in people with dementia, who face challenges when completing health-related questionnaires. To improve feasibility for our participants, we introduced necessary flexibility into our administration of the HHIE. Whenever possible, we encouraged the older person with dementia to be actively involved in the completion of the HHIE—whether independently or together with a family caregiver. However, when necessary, family caregivers completed the HHIE for the individual with dementia based on their own perception of the person with dementia’s hearing difficulty. HHIE scores were only used descriptively and were not used to determine eligibility or classify participants, given the lack of validation in dementia populations.

We used the Lawton-Brody IADL Scale to quantify independent living skills and the level of assistance participants with dementia require for completing IADL tasks that involve self-management skills that may be relevant to OTC hearing aid use [47]. The Lawton-Brody scale includes eight questions covering the following functional domains: using the telephone, shopping, food preparation, housekeeping, laundry, transportation, medication management, and finances. Each item is rated dichotomously as able (one point) or less able (0 points). Points are summed to give a total score, and dependence is defined as a score <8 points for women and <5 points for men due to differences in traditional gender roles [48]. The questionnaire may be administered to either persons with dementia or family caregivers. In this study, we requested that a family caregiver complete the Lawton-Brody whenever possible, as people with dementia may be hesitant to report functional deficiencies in the home. If a family caregiver was unavailable, the person with dementia completed the questionnaire independently.

### Interviews

Participants completed an individual interview conducted via secure Zoom (Zoom Communications) videoconferencing. Interviews followed a semistructured interview guide consisting of open-ended questions designed to gather interest-holder-perceived facilitators and barriers to OTC hearing aids for community-dwelling older people with co-occurring early to midstage dementia and hearing loss (Multimedia Appendix 1). Consistent with semistructured qualitative interviewing techniques, each interview followed a set of predefined questions; however, the interviewer followed the participant’s lead in conversation and asked tailored follow-up questions as appropriate. Participants were allowed and encouraged to explore any issue of interest to them at the intersection of hearing loss, communication, hearing aid use, dementia, and dementia caregiving.

Each interview began with a brief introduction to OTC hearing aids and their main differences from prescription hearing aids, focusing on factual distinctions between regulatory categories to avoid biasing participant responses. Subsequent interview questions followed the main steps of the OTC hearing aid consumer pathway, eliciting participant views on determining OTC hearing aid candidacy, purchasing OTC hearing aids, configuring OTC hearing aids, assessing OTC hearing aid benefits, and using, maintaining, and troubleshooting OTC hearing aids. The interview guide was adapted for each participant group but maintained the same overall structure and sequencing of topics to ensure the similarity of interview content across participants. Interview guides for each participant group are included in the Multimedia Appendix 1.

Several accommodations were needed for individuals with dementia. For most participants with dementia, a family member assisted with Zoom and other study logistics. In these cases, family caregivers were instructed to allow the person with dementia to answer interview questions independently and to assist only as necessary to help the participant understand study directions and remain on task. Additionally, interview questions were modified as needed to ensure understandability for participants with dementia, who present with individual differences in cognitive and language ability not well predicted by their self- or caregiver-reported dementia status and quantitative measures of cognitive functioning. However, even with question modifications, participants with dementia often could not answer all interview questions. In these cases, participants with dementia were included in the study if they demonstrated an understanding of the definition of OTC hearing aids through their responses and engaged in the interview by providing independent and relevant answers to a subset of the interview questions.

The interviews were conducted by the first author, a female audiologist (AuD) who, at the time of the study, was a doctoral candidate and clinical researcher with formal training and experience in clinical audiology, qualitative interviewing, and dementia-related research. No prior relationship was established between the interviewer and any participant before study commencement. Participants were informed that the interviewer was an audiologist and researcher conducting a study on perspectives regarding hearing aid use and dementia. To minimize potential bias, participants were not informed that the study focused specifically on OTC hearing aids until the interview. The interviewer has a professional interest in improving hearing care for people living with dementia and remained attentive to ensuring that participants’ perspectives guided the interviews.

All interviews were audio-recorded, transcribed verbatim, and anonymized. Interviews typically lasted between 30 and 60 minutes each. No repeat interviews were conducted. Field notes were not taken during or after interviews. The University of Minnesota Institutional Review Board approved the interview guide and all study procedures.

### Data Analysis

Interview transcripts were reviewed and analyzed in NVivo 12 (QSR International Pty Ltd, 2022). We used reflexive thematic analysis as our methodological orientation, following Braun and Clarke’s [49] six steps for thematic analysis: (1) familiarization, (2) generation of initial codes, (3) search for themes, (4) review, (5) name and define themes, and (6) write-up. First, 2 qualitative coders independently read the deidentified transcripts to familiarize themselves with participant responses (step 1). Then, the coders convened to discuss emerging patterns, using an inductive approach, and generate an initial coding framework (step 2). The coding framework considered all participants together—rather than partitioning them into their interest-holder groups—to facilitate subsequent comparison and contrast of participant perspectives. Next, the 2 coders individually coded one randomly selected transcript from each participant group and convened to compare interpretations and refine/clarify the coding framework. The first author coded all remaining transcripts, with the second coder double-coding 20% of transcripts to ensure consistency of code application. During this process, regular meetings were held to review codes, and disagreements were resolved through discussion. After coding was complete, the coders collated codes into themes and subthemes (step 3), which were subsequently reviewed, named, and defined by the research team (steps 4 and 5). The resulting themes were grouped under 2 main headings, which frame the results narrative (step 6): (1) facilitators or advantages of OTC hearing aid use for community-dwelling older adults with dementia, and (2) barriers to or disadvantages of OTC hearing aid use in these individuals. Formal data saturation thresholds were not prespecified; however, no substantially new themes emerged during later analysis, suggesting that saturation had been reached. Transcripts and themes were not returned to participants for comment, correction, or feedback.

## Results

### Participant Characteristics

The study included a total of 45 participants split equally between the 3 interest-holder groups (15 each). No eligible participants declined to participate, and no enrolled participants withdrew from the study. Most family caregivers and direct care professionals were female, consistent with published demographics of both groups [50-52]. Family caregivers had a mean age of 62.7 (range 32-79, SD 13.1) years, and just over half had experience assisting their care recipient with hearing aids (n=8). Direct care professionals had a mean age of 46.7 (range 26-74, SD 14) years and all but one (n=14) reported assisting their patients with routine hearing aid use, such as insertion and removal, changing batteries/charging hearing aids, and routine cleaning/care. In contrast with family caregivers and direct care professionals, people with dementia were predominantly male. This outcome was somewhat unexpected, as Alzheimer or related dementia disproportionately affects women; however, it is also well documented that men are more likely than women to have hearing loss [53]. Participants with dementia had a mean age of 82.1 (range 68-95, SD 7.7) years, and most were current hearing aid users (n=11). Detailed participant characteristics are provided in Tables 1-3.

**Table 1:** Participant characteristics for persons with dementia (N=15).

Characteristic			Values
**Sex, n (%)**			
	Female	4 (27)	
	Male	11 (73)	
Age (years), mean (SD)			82.1 (7.7)
**Education, n (%)**			
	Technical	2 (13)	
	High School	3 (20)	
	Some college	2 (13)	
	Undergraduate	3 (20)	
	Graduate	5 (33)	
**Marital status, n (%)**			
	Married	9 (60)	
	Widowed	5 (33)	
	Divorced	1 (7)	
**Lives alone, n (%)**			
	Yes	4 (27)	
	No	11 (73)	
**Hearing aid user, n (%)**			
	Yes	11 (73)	
	No	4 (27)	
TICS-m^a^, mean (SD)			17.2 (6.2)
HHIE^b^, mean (SD)			36 (21.4)
Lawton-Brody IADL^c^, mean (SD)			2.27 (2.2)

^a^Education-adjusted Telephone Interview for Cognitive Status–modified (TICS-m) calculated using corrections from Knopman et al [44].

^b^Hearing Handicap Inventory for the Elderly (HHIE); completed independently by the person with dementia (n=3), by a family caregiver (n=5), or jointly (n=7).

^c^Lawton-Brody Instrumental Activities of Daily Living (IADL) Scale; completed by a family caregiver (n=14) or the person with dementia (n=1).

**Table 2:** Participant characteristics for family caregivers (N=15).

Characteristic			Values
**Sex, n (%)**			
	Female	14 (93)	
	Male	1 (7)	
Age (years), mean (SD)			62.7 (13.1)
**Education, n (%)**			
	Some college	1 (7)	
	Undergraduate	5 (33)	
	Graduate	9 (60)	
**Relationship to care recipient, n (%)**			
	Spouse	8 (53)	
	Child	6 (40)	
	Grandchild	1 (7)	
**Lives with care recipient, n (%)**			
	Yes	11 (73)	
	No	4 (27)	
**Care recipient uses hearing aids, n (%)**			
	Yes	8 (53)	
	No	7 (47)	

**Table 3:** Participant characteristics for direct care professionals (N=15).

Characteristic		Values
**Sex, n (%)**		
	Female	14 (93)
	Male	1 (7)
Age (years), mean (SD)		46.7 (14.0)
**Education, n (%)**		
	Undergraduate	9 (60)
	Graduate	6 (40)
**Occupation, n (%)**		
	Registered nurse	8 (53)
	Physical therapist	2 (13)
	Certified nursing assistant	1 (7)
	Clinical mental health counselor	1 (7)
	Nurse practitioner	1 (7)
	Occupational therapy assistant	1 (7)
	Social worker	1 (7)
**Years of experience, n (%)**		
	1-5	3 (20)
	6-10	4 (27)
	11-15	3 (20)
	16-20	2 (13)
	21+	3 (20)

### Interview Themes

In the following sections, we present key themes and subthemes generated from the thematic analysis process described above. Table 4 outlines these themes and provides exemplar quotes for each.

**Table 4:** Themes and descriptions.

Theme			Description
**Facilitators**			
	Accessibility	Participants emphasized the ease of obtaining OTC^a^ hearing aids and the benefit of eliminating the need for recurrent office visits for hearing aid fittings and adjustments.	
	Affordability	Participants appreciated the lower cost of OTC hearing aids, with some highlighting their potential to offer better overall value for individuals with dementia.	
	Autonomy and control	Participants valued the freedom, flexibility, and autonomy to try OTC hearing aids independently, noting that this approach could also reduce stigma associated with traditional hearing care.	
**Barriers**			
	Mistrust of OTC hearing aids	Participants expressed skepticism about the quality, customization, and ethics of OTC hearing aids, particularly regarding their ability to meet the needs of people with dementia.	
	Assessing OTC hearing aid candidacy in people with dementia	Participants were concerned about the ability of people with dementia to accurately self-classify their hearing loss and questioned the reliability of family caregivers’ assessments without professional guidance.	
	Caregiver-facilitated programming and adjustment	Participants doubted whether family caregivers could effectively program and adjust OTC hearing aids without professional support.	
	Assessing the effectiveness of OTC hearing aids	Evaluating the benefit of OTC hearing aids was seen as challenging, particularly when distinguishing the effects of hearing loss versus cognitive decline on communication.	
	Ongoing OTC hearing aid use and caregiver burden/burnout	Family caregivers and direct care professionals expressed concerns about the additional responsibilities OTC hearing aids might place on family caregivers, particularly given the challenges of caregiver burden and burnout.	

^a^OTC: over-the-counter.

### Facilitators of OTC Hearing Aid Use in People With Dementia

#### Overview

Across the 3 interest-holder groups, participants identified important facilitators/advantages of OTC hearing aid use for community-dwelling older people living with co-occurring dementia and hearing loss. Under this heading, we identified 3 main themes described below.

##### Facilitator Theme #1: Accessibility

Consistent with the FDA’s rationale for OTC hearing aids, participants in all 3 groups expressed optimism that OTC hearing aids can increase access to hearing aids for older people with dementia. Improved accessibility, participants noted, benefits many older adults with hearing loss and not solely those with dementia. However, participants also explained that better access to amplification has particularly high salience in the dementia caregiving context. Family caregivers and direct care professionals highlighted the advantage of eliminating the need for recurrent office visits to fit and adjust prescription hearing aids. Both family caregivers and care professionals emphasized that dementia caregiving is “consuming” [caregiver, wife, age (years) in the range of 60s], involving round-the-clock care, including providing transportation to and from frequent health care appointments for dementia and its complications. From this perspective, a subtheme emerged that family caregivers may view recurrent hearing aid office appointments as “burdensome” [care professional, female, age (years) in the range of 50s], a “struggle” [care professional, female, age (years) in the range of 40s], and “too hard.” [caregiver, daughter, age (years) in the range of 60s] As a result, many family caregivers are “apt to not show up for the appointments or not do it at all.” [care professional, female, age (years) in the range of 50s] Conversely, participants across groups viewed accessing OTC hearing aids as a fast, easy, and convenient alternative. As one person with dementia remarked, OTC hearing aids are “probably the easiest way to start.” [person with dementia, male, age (years) in the range of 90s]

Participants explained that the time and travel involved in prescription hearing aid fittings are not only inconvenient but can also have financial, social, and emotional ramifications. Family caregivers and care professionals explained that people with dementia spend large amounts of time seeing health care providers. This “time investment” [care professional, female, age (years) in the range of 40s] is costly for individuals with dementia and their family caregivers. For caregivers, the cost is often measured in terms of the financial and employment consequences of taking time off work to transport care recipients with dementia to health care appointments.

As one family caregiver remarked:

If I have to schedule with the audiologist … it requires me to take time off work and then travel there, so it’s costly in a number of ways, you know?

caregiver, daughter, age (years) in the range of 60s

For people with dementia, the cost is measured indirectly in lost time for social engagement and other activities. As one family caregiver commented:

Going to the audiologist is not the most exciting thing for her [care recipient]. I mean, she’s kind of missing out on other activities and social opportunities and things if she’s having to go to a lot of appointments.

caregiver, granddaughter, age (years) in the range of 30s

Many family caregivers and care professionals also raised concerns about the emotional impact of health care appointments on people with dementia. They shared that health care visits, including audiology appointments, can be difficult experiences for people with dementia, who may struggle to recognize, navigate, and understand large medical buildings and unfamiliar health care providers. One care professional commented on this challenge for prescription hearing care and shared how OTC hearing aids might help:

As they get further into the dementia, going to a new doctor or a place that’s different—like having their hearing checked—being in that office situation can be scary for them. So if it’s [hearing aid fitting] something they could do at home with a loved one, that would definitely be helpful for them.

care professional, female, age (years) in the range of 40s

Similarly, one care professional observed:

Sometimes the medical buildings are hard to navigate… and that can be overwhelming for people with dementia. So, I think it’d be easier being able to go to the pharmacy up the street or something they’re familiar with to buy hearing aids.

care professional, female, age (years) in the range of 20s

Put simply, one person with dementia anticipated that if they decided to purchase OTC hearing aids, “it would be easy to get there.” [person with dementia, female, age (years) in the range of 80s]

##### Facilitator Theme #2: Affordability

Participants in all 3 groups identified affordability as a key advantage or facilitator of OTC hearing aid use in people with dementia. Indeed, participants felt that more affordable hearing aids benefit all older adults, including those with dementia. However, perceptions of cost took on greater nuance in the context of dementia caregiving. Rather than emphasizing cost alone, many family caregivers spoke about value (ie, expected benefit/worth relative to price). The value subtheme was exemplified by a subset of family caregivers who shared that although they could afford prescription hearing aids, they questioned their benefit for people with dementia. Several family caregivers shared that the progression of dementia seemed to make their care recipient content to avoid conversation. They wondered if their relative was interested in hearing better and participating in conversations—and, in turn, whether expensive hearing aids were worth the cost. For example, one family caregiver reflected:

The interesting thing about Alzheimer’s is like … he’s happy in his own little world and so he likes being with people—but no, he doesn’t necessarily want to talk … or be part of the conversation. That’s not necessarily important to him right now. So, it makes it like … what’s it gonna cost me? What’s gonna be the benefit?

caregiver, wife, age (years) in the range of 60s

Family caregivers also raised concerns about whether people with dementia can take full advantage of the more expensive advanced features and professional fitting services that come with prescription hearing aids. Many noted that Bluetooth technologies, smartphone apps, and other hearing aid accessories are “technically more challenging than people with dementia can work with” [caregiver, daughter, age (years) in the range of 70s] and would, therefore, go unused. Several family caregivers also questioned whether people with dementia can provide sufficiently meaningful, reliable input to justify the added cost of professional hearing aid adjustments. As one family caregiver said,

It might be better that they go to an audiologist and really get the exact fit for what they need, but my husband is at the point right now where he can’t tell you what the exact right fit is, so that’s not applicable to him.

caregiver, wife, age (years) in the range of 60s

Finally, family caregivers expressed concern about the expense of prescription hearing aids relative to their high risk of being lost or damaged by people with dementia. One family caregiver explained:

A lot of dementia patients lose things. So honestly, if I think he’s [care recipient] gonna lose his hearing aids again, I’m gonna go with the cheapest ones around because they’re only gonna last until the next time he loses ‘em.

caregiver, wife, age (years) in the range of 60s

Importantly, many family caregivers felt that lower-cost OTC hearing aids could mitigate their concerns about the value of hearing aids for people with dementia. For example, one family caregiver remarked:

I would hate to get those professional hearing aids and either they’re lost or damaged, or she can't utilize them in the way they need to be. Yeah, I could swallow it a little bit easier if it’s a thousand-dollar OTC hearing aids.

caregiver, daughter, age (years) in the range of 50s

This and other family caregiver responses suggest OTC hearing aids may help shift caregivers’ value calculations in favor of purchasing and trying amplification.

##### Facilitator Theme #3: Autonomy and Control

Another facilitator theme emerged around control—or the freedom, flexibility, and autonomy to efficiently try OTC hearing aids and make decisions about them. Across all 3 groups, several participants saw OTC hearing aids as an opportunity for people with dementia and their family caregivers to “scope out hearing aids for themselves” [care professional, female, age (years) in the range of 50s] and more rapidly evaluate their benefits. As one family caregiver put it:

I think that, in general, to have a fast way … to just get a hearing aid to your person and see if it works is a wonderful, wonderful thing.

caregiver, wife, age (years) in the range of 60s

Relatedly, many family caregivers and care professionals highlighted the advantage of quickly gauging the likelihood of hearing aid tolerance and acceptance in people with dementia. One care professional explained:

With OTC hearing aids, they [family caregivers] could probably get a general idea of if they’re [care recipients] gonna be compliant with hearing aids right away.

care professional, female, age (years) in the range of 30s

Additionally, a prominent subtheme emerged around stigma. Direct care professionals stressed the deep-seated stigma and “embarrassment” [care professional, female, age (years) in the range of 30s] that accompany a diagnosis of age-related hearing loss. Receiving this diagnosis, participants explained, means a person is “labeled as old now” [care professional, female, age (years) in the range of 40s] and—especially for community-dwelling persons with dementia—may invoke fear of losing independence. As a result, those with dementia may avoid seeing a hearing care professional altogether. Several care professionals felt OTC hearing aids could help circumvent stigma by giving older adults with dementia and their caregivers the autonomy and control to try hearing aids “on the sly without actually having it in their medical records.” [care professional, female, age (years) in the range of 50s] One care professional explained:

They [community-dwelling people with dementia] don’t want hearing loss to be another thing that makes them feel incapable of taking care of themselves … They’re at home. They don’t want to move, so then they don’t want to bring attention to their hearing.

care professional, female, age (years) in the range of 20s

Many care professionals and family caregivers felt that some people with dementia would be more open to OTC hearing aids, which allow them to try amplification without “admitting to a loss.” [care professional, female, age (years) in the range of 50s] or “involving other people.” [person with dementia, male, age (years) in the range of 90s] As one family caregiver reflected:

It might be easier for him [care recipient] to accept not going in to see a doctor … There might be an advantage that I could at least introduce him to hearing aids this way.

caregiver, wife, age (years) in the range of 60s

### Barriers to OTC Hearing Aid Use in People With Dementia

#### Overview

Although participants described meaningful potential facilitators/advantages of OTC hearing aids for people with dementia, they also identified several unaddressed barriers or disadvantages. Below, we describe 5 main barrier themes.

##### Barrier Theme #1: Mistrust of OTC Hearing Aids

Across all 3 groups, many participants expressed a general mistrust of OTC hearing aids. This theme emerged from a variety of statements revealing apprehension, hesitance, and skepticism about the quality and effectiveness of OTC hearing aids and their associated support services. Specifically, some participants raised concerns that OTC hearing aids may lack sufficient customization of their frequency responses to match individual hearing losses. These participants viewed OTC hearing aids as simple “amplifiers” [caregiver, wife, age (years) in the range of 70s; care professional, female, age (years) in the range of 60s; person with dementia, male, age (years) in the range of 70s], which they believed to be too “generic” [caregiver, daughter, age (years) in the range of 60s; caregiver, wife, age (years) in the range of 60s] for people with hearing loss—especially those with dementia whose hearing loss is further complicated by the effects of cognitive decline. A participant with dementia commented:

I am suspicious of [OTC hearing aids] … because it’s not talking about my hearing in each ear. It is trying to be an amplifier. I got a friend who doesn’t wanna pay for [prescription hearing aids]. He says, ‘I’ll a buy a set of those [OTC hearing aids].’ And I said, ‘Good luck. You’re gonna waste your money.’

person with dementia, male, age (years) in the range of 70s

A subtheme emerged in which participants questioned the competence, training, and expertise of OTC hearing aid vendors and customer support staff. Across groups, participants overwhelmingly endorsed the need for readily available, knowledgeable, and trustworthy technical support to facilitate OTC hearing aid use in people with dementia. At the same time, participants expressed uncertainty that OTC hearing aids would include “any technical assistance that you can count on.” [caregiver, daughter, age (years) in the range of 70s] Specifically, participants raised concerns that the people who sell and service OTC hearing aids may have insufficient training and experience in hearing loss and hearing aids—and, therefore, might not provide helpful, actionable recommendations. This sentiment was especially prominent among people with dementia. As one participant with dementia remarked:

Well, not being familiar with buying over the counter, the clerk would not know how to explain properly how to do it versus a doctor at the clinic.

person with dementia, male, age (years) in the range of 80s

Along the same lines, another participant with dementia said:

I don’t know if it [OTC hearing aids] would be easier. ‘Cause sometimes they don’t know exactly what you need or to tell you how to use it.

person with dementia, male, age (years) in the range of 70s

Participants with dementia, in particular, expressed doubt about the ethics and morality of the people who sell and service OTC hearing aids. They described feeling skeptical—“iffy” [person with dementia, male, age (years) in the range of 70s]—about the veracity of the “smiley ads” [person with dementia, male, age (years) in the range of 70s] for OTC hearing aids and questioned their underlying motives. Many participants with dementia felt that OTC hearing aids only emphasized sales and profits at the expense of older adults living on fixed incomes. As one participant with dementia explained, “you could be fooled by somebody that’s just trying to make some money.” [person with dementia, female, age (years) in the range of 80s] Another participant with dementia asked:

Who’s on the other side of the counter? … I wouldn’t even consider that. Not at all. To me that would be like a crapshoot.

person with dementia, male, age (years) in the range of 80s

##### Barrier Theme #2: Assessing OTC Hearing Aid Candidacy in People With Dementia

An important barrier theme emerged around assessing OTC hearing aid candidacy in people with dementia. According to FDA regulations, OTC hearing aids are intended for adults with perceived mild-to-moderate hearing loss*.* However, many care professionals and family caregivers questioned the accuracy and reliability of perceived hearing loss in people with dementia. Both participant groups overwhelmingly agreed that people with dementia cannot accurately self-classify their degree of hearing loss. Specifically, a subtheme emerged surrounding the memory demands of self-assessing one’s hearing loss. As several family caregivers and care professionals noted, age-related hearing loss gradually “creeps up” [care professional, female, age (years) in the range of 50s] on older adults, thus requiring them to recall and reflect on their hearing experiences over both long and short periods of time. Many people with dementia, participants explained, do not have the memory and cognitive capacity to remember and compare their listening experiences. As a result, family caregivers and care professionals were doubtful that people with dementia could accurately describe their hearing loss progression and categorize its severity relative to others’ hearing ability. One care professional explained:

I think people with dementia could identify profound hearing loss—that they can’t hear. But anything less, you add the complication of dementia. What are they not remembering? To say mild or moderate versus getting moderate to severe, they wouldn’t know.

care professional, female, age (years) in the range of 40s

During semistructured interviews, many participants with dementia struggled to answer questions about determining their own candidacy for OTC hearing aids. However, those who could respond shared a similar opinion. For example, when asked if he was confident in self-classifying his degree of hearing loss, one participant with dementia responded:

Probably not because it comes on so gradually. And after you’ve been living with the condition, you sort of just accept that’s the way your hearing is … It gets integrated into your life.

person with dementia, male, age (years) in the range of 80s

Family caregivers and care professionals agreed that in many cases, family members would be responsible for determining OTC hearing aid candidacy for people with dementia. However, family caregivers lacked confidence that they could accurately classify their care recipient’s degree of hearing loss without a professional hearing examination. Here, a notable subtheme emerged regarding the challenge of separating the effects of cognitive decline/dementia and hearing loss on communication in people with dementia. Many family caregivers shared that they were unsure if they were observing symptoms of cognitive decline or hearing loss. As one family caregiver reflected:

I don’t know if she’s [care recipient] not holding that conversation because she doesn’t have the capacity and the wherewithal to hold that conversation, or if she’s really not hearing the other person speak.

caregiver, wife, age (years) in the range of 70s

Given the challenges described above, participants in all 3 groups felt it was important that people with dementia have a professional hearing examination regardless of their or their family’s interest in OTC hearing aids. A care professional commented:

Even though it’s over the counter, I still feel like there would be a need for… some type of evaluation and referral from a specialist. Not just that the caregiver says, ‘Well, I’ve been talking recently, and they’ve not been responding back.’

care professional, female age (years) in the range of 30s

Similarly, one participant with dementia explained:

People try to guess their hearing loss. But you have to use technology to figure it out. You have to sit in that box. And they do the little noises back and forth, and then when you’re done each ear is tested for its own hearing.

person with dementia, male, age (years) in the range of 70s

##### Barrier Theme #3: Caregiver-Facilitated Programming and Adjustment

Participants in all 3 groups overwhelmingly expressed uncertainty and trepidation about the process of programming and adjusting OTC hearing aids for people with dementia. First and foremost, there was strong agreement that by and large, people with dementia cannot independently self-program and customize OTC hearing aids—regardless of the specific self-programming or fitting process used. Indeed, many family caregivers and care professionals pointed out that people with dementia—even in the early stages of the disease—often need help using everyday technologies such as smartphones, televisions, computers, and tablets. Similarly, participants anticipated that family caregivers would play a significant role in programming and adjusting OTC hearing aids for people with dementia.

However, participants questioned whether family caregivers possess sufficient knowledge, skills, and experiences to program OTC hearing aids for care recipients with dementia. Some participants highlighted that family caregivers are not “educated in hearing” [person with dementia, female, age (years) in the range of 80s], while others stressed that many dementia caregivers are themselves older adults who may face barriers to learning and using new technologies. As one family caregiver reflected:

When you think about it, a lot of the people that need hearing aids are old, right? And most people my age do not understand technology very well and they wouldn’t be able to program hearing aids for their spouse.

caregiver, wife, age (years) in the range of 70s

Most participants expressed a preference for and confidence in professional hearing aid programming and adjustments. Across groups, participants emphasized the complexity of human hearing and hearing aids, along with the education and training of licensed hearing care professionals— “there’s a lot to hearing,” [caregiver, daughter, age (years) in the range of 60s] one family caregiver commented. Participants especially valued professional expertise in programming and adjusting hearing aids based on an individual’s audiometric thresholds, behavioral responses, and preferences. Specifically, several family caregivers and participants with dementia viewed hearing care professionals as uniquely attuned to “the correct questions to ask” [person with dementia, female, age (years) in the range of 80s], a person with hearing loss—including those with dementia—when tailoring and optimizing an individual’s hearing aid settings. As one family caregiver explained:

I’d prefer [care recipient’s hearing aids] be programmed by a professional who asks the questions and compares, ‘How is it now? How is it not now?’ Whether that same quality of observation could happen with me doing it—I don’t know.

caregiver, husband, age (years) in the range of 70s

Similarly, one participant with dementia shared:

I’d rather go to someone that [programmed hearing aids] all the time…. Because they had probably done a lot of work with people with hearing aids, so they would know a lot of questions to ask about how I was hearing.

person with dementia, female, age (years) in the range of 80s

In particular, family caregivers were unsure if they would ask the right questions of their care recipient when programming OTC hearing aids. This concern was magnified by a subtheme regarding the challenge of obtaining reliable self-reports of sensory symptoms from people with dementia. Both family caregivers and direct care professionals emphasized that people with dementia lose the “self-reflective ability” [caregiver, wife, age (years) in the range of 70s] and “correct words” [care professional, female, age (years) in the range of 40s] to clearly identify and describe bodily and sensory sensations. Consequently, participants expressed concern that family caregivers would not be able to elicit and properly interpret feedback from their care recipient to facilitate effective OTC hearing aid programming. As a result, caregivers anticipated that they “[would] not trust” [caregiver, wife, age (years) in the range of 60s] and “would not feel confident” [caregiver, daughter, age (years) in the range of 60s] in their “amateur” [caregiver, husband, age (years) in the range of 70s] programming results. Several participants with dementia echoed this sentiment, one of whom explained:

She’s [family caregiver] good with a lot of things, but I don’t know that she’s an expert in that [OTC hearing aid programming]. She is not educated in hearing, and I believe that you have to be educated with the body in order to work with improving hearing.

person with dementia, female, age (years) in the range of 80s

One family caregiver summarized her concerns about using an OTC hearing aid for her care recipient:

I think [programming is] the major disadvantage, not knowing if you’ve programmed it for the best quality of hearing, especially for somebody else

caregiver, daughter, age (years) in the range of 60s

##### Barrier Theme #4: Assessing the Effectiveness of OTC Hearing Aids

Following OTC hearing aid programming, participants raised concerns about their ability to assess the effectiveness of OTC hearing aids in people with dementia. Across groups, most participants felt they could determine if OTC hearing aids were helpful to some degree—but they questioned their ability to judge whether the devices had been “optimized” [caregiver, wife, age (years) in the range of 60s] or had achieved a person with dementia’s full hearing potential. Here, participants sought assurance that OTC hearing aids had delivered “the very best benefit” [caregiver, wife, age (years) in the range of 70s] for speech understanding and communication. However, many participants felt such assurance could only be obtained by seeing a hearing care professional for a prescription hearing aid fitting. Participants in all 3 groups expressed trust and confidence in a hearing care professional’s ability to ensure hearing aids are “correct… and fit beautifully enough for [people with dementia] to hear.” [care professional, female, age (years) in the range of 30s] As one participant with dementia explained when discussing hearing aid programming:

Since I’m a perfectionist, I wanna talk to somebody who is in the [hearing aid] business.

person with dementia, female, age (years) in the range of 80s

Without the assistance of a professional, participants in all 3 groups were unsure how they would assess and quantify the effectiveness of OTC hearing aids. Specifically, family caregivers and direct care professionals noted two prominent subthemes described earlier: (1) the challenge of separating the effects of cognitive decline/dementia and hearing loss on communication in people with dementia, and (2) the challenge of obtaining a reliable self-report of sensory symptoms from people with dementia. Together, these 2 challenges made family caregivers and direct care professionals apprehensive about evaluating the amount and extent of OTC hearing aid benefit in people with dementia—“it’s tricky because you’re also observing cognition,” [caregiver, wife, age (years) in the range of 70s] one family caregiver noted. Another family caregiver reflected:

When you have cognitive decline, all parts of the brain are dying literally and not functioning as well. It takes [care recipient] longer to process things that are said. And I might have less confidence in [care recipient’s] ability to describe it [hearing aid performance].

caregiver, wife, age (years) in the range of 70s

Most participants emphasized the importance of programming OTC hearing aids for optimal speech understanding and communication; however, several family caregivers and direct care professionals raised concerns about the possibility of sensory agitation from poorly fit OTC hearing aids. Responses in this subtheme emphasized the sensitivity of people with dementia to changes in their environment and sensory inputs. Namely, participants wondered if suboptimal OTC hearing aids could produce uncomfortable sound quality or acoustic feedback that might make a person with dementia startled, agitated, frustrated, or withdrawn. Participants raised specific concerns about “loud sounds,” [caregiver, daughter, age (years) in the range of 40s] “echoing,” [care professional, female, age (years) in the range of 70s] “ringing,” [care professional, female, age (years) in the range of 20s] and “buzzing” [care professional, female, age (years) in the range of 50s] as likely sources of sensory agitation from hearing aids that might be overlooked or poorly managed in OTC hearing aids. Notably, participants lacked confidence in both people with dementia and their family caregivers to accurately identify, assess, and resolve these issues. One care professional explained:

If they [people with dementia] can't tell you that the [hearing aid] volume is too loud, it could be echoing and stuff. You never would know that because they have dementia, and they just won't talk at all because the sound in their ear is not correct… like a buzz or a ringing.

care professional, female, age (years) in the range of 70s

Adding further challenge, caregivers and care professionals felt they might have limited time to identify and resolve OTC hearing aid issues before their care recipient might become frustrated or uncooperative. Here, a subtheme emerged for the tendency of people with dementia to “give up early” [care professional, female, age (years) in the range of 40s] on new technologies. Several participants stressed that if the effectiveness and benefit of OTC hearing aids were not quickly apparent, a person with dementia might abandon the devices altogether. One family caregiver explained:

My husband [care recipient] would expect me to be the expert on it [OTC hearing aids], and then if it didn't work, he'd get cranky at me, and he might give up.

caregiver, wife, age (years) in the range of 60s

A participant with dementia shared his own expectations for immediate OTC hearing aid benefit:

If it's [OTC hearing aids] wrong, I wouldn't have any idea what to do. It's so simple…. You put it [hearing aid] in your ear and press the button. If it doesn’t work… that isn’t right.

person with dementia, male, age (years) in the range of 80s

##### Barrier Theme #5: Ongoing OTC Hearing Aid Use and Caregiver Burden/Burnout

Family caregivers and direct care professionals agreed that the challenges of OTC hearing aid use in people with dementia extend beyond the initial purchase and programming. Indeed, both groups stressed that OTC hearing aid use is an ongoing commitment involving daily responsibilities for hearing aid use, care, maintenance, and troubleshooting. Across groups, participants had differing views on the amount and type of hearing aid tasks a person with dementia could complete independently, reflecting individual differences between care recipients. Nevertheless, participants concurred that family caregivers would need to assume ultimate responsibility for overseeing and managing continued OTC hearing aid use for a person with dementia—especially as their cognitive and functional abilities further declined. This responsibility emerged as a significant concern. Nearly all family caregivers emphasized their multiple competing caregiving responsibilities, with many describing themselves as overwhelmed, overloaded, and burned out. For this reason, many family caregivers questioned their patience and capacity for self-learning and implementing OTC hearing aid tasks without the assistance of a hearing care professional. “The caregiver needs to be supported and educated to be successful… that’s pretty important in dementia care,” one family caregiver explained [caregiver, daughter, age (years) in the range of 60s]. Another family caregiver shared her initial reaction to learning about OTC hearing aids on television:

What I thought about [OTC hearing aids] is, I would find it challenging to figure out. I have to say as a caregiver and being kind of burned out that I’m not as confident doing things as I used to be. I mean, I’m aging, too, but I think it has more to do with being overwhelmed and burned out. It’s harder for me to learn things right now that I would normally be able to manage.

caregiver, daughter, age (years) in the range of 60s

Another family caregiver echoed this perspective:

I can figure out technology without any problem…but I get very frustrated very easily when I have to do anything on my own. It’s not that I can’t do it, it’s just that you have so much on your plate that if somebody puts one more thing on your plate, it puts you on overload.

caregiver, wife, age (years) in the range of 70s

Family caregivers were unsure if the challenges of long-term OTC hearing aid use would be worth their effort. Here, family caregivers returned to the subtheme of value discussed earlier; however, they augmented their previous calculation of price versus expected hearing aid benefit with a new calculation of their caregiving work (cost) versus observed hearing aid benefit. In this context, family caregivers anticipated that their work would be substantial while OTC hearing aid effectiveness would be uncertain or unclear (returning to an earlier barrier theme— see Barrier #4). Furthermore, family caregivers questioned whether their care recipient would be cooperative, compliant, or equally invested in using OTC hearing aids. As one family caregiver described:

It’s [OTC hearing aid use] one more thing to do. It’s questionable benefit…. And unclear how much cooperation you’re gonna get from your partner. And you learn where to fight your battles.

caregiver, husband, age (years) in the range of 70s

Another family caregiver elaborated:

It’s always gonna be how much work is it versus the benefit. That’s always gonna be, as a caregiver, the scale you’re in.

caregiver, wife, age (years) in the range of 60s

Finally, when considering whether she would use OTC hearing aids with her spouse, who has dementia, one family caregiver shared her competing priorities as a dementia caregiver: “I need to make my husband’s [care recipient] life easier, but I also need to make our lives easier.” [caregiver, wife, age (years) in the range of 60s].

## Discussion

### Principal Findings

This qualitative study explored the feasibility and acceptability of now widely available OTC hearing aids for community-dwelling older people with dementia and their family caregivers. We approached this question from the perspectives of key dementia-care interest-holders via semistructured interviews with individuals from three interest-holder groups: (1) community-dwelling older adults with co-occurring dementia and hearing difficulty, (2) family caregivers of community-dwelling older adults with co-occurring dementia and hearing difficulty, and (3) geriatric direct care professionals. Using thematic analysis, we identified and described specific facilitators and barriers to OTC hearing aid use in community-dwelling older people with dementia. Below, we discuss our findings and their clinical implications.

### Facilitators of OTC Hearing Aid Use in People With Dementia

Facilitators or advantages of OTC hearing aid use included accessibility, affordability, and autonomy/control. While these benefits extend to older adults in general, our participants highlighted their specific importance within the dementia caregiving context. In terms of accessibility, participants endorsed the benefit of OTC hearing aids in eliminating the recurrent in-person appointments typically required for prescription hearing aid fitting and adjustments. Participants felt that removing the need for in-person appointments could make hearing health care more feasible and palatable for dementia caregivers and their care recipients. Additionally, participants appreciated the affordability of OTC hearing aids. However, family caregivers tended to emphasize value considerations rather than cost. Here, family caregivers viewed OTC hearing aids as a potentially better value than prescription hearing aids when considering their care recipients’ simple listening environments, low technology proficiency, and high proclivity for losing/damaging hearing aids and other technologies. Finally, participants also identified the benefits of OTC hearing aids in the realm of autonomy and control. Across interest-holder groups, participants appreciated the autonomy and flexibility OTC hearing aids offer for deciding when, where, and how to try hearing aids for people with dementia. Participants viewed this enhanced control as particularly important within the context of stigma, noting that the combined stigmas of dementia and hearing loss may prevent individuals with dementia from seeking or agreeing to formal hearing testing or prescription hearing aids. Participants in all 3 groups agreed that OTC hearing aids could offer a more discreet alternative by allowing people with dementia to circumvent formal hearing testing and documentation of prescription hearing aid use.

These findings generally align with the larger hearing aid and dementia caregiving literature. Regarding accessibility, the benefits of OTC hearing aids are evident. Studies show family dementia caregivers bear heavy responsibility for transporting their care recipient to health care appointments, which can contribute to caregiving-related emotional, psychological, financial, and physical burden [54,55]. Furthermore, health care appointments may—as our participants discussed—cause emotional and physical distress for people with dementia, particularly when health care providers are not specifically trained in dementia [56]. Given these considerations, our participants’ desire to avoid recurrent in-person hearing aid appointments is understandable and may constitute a significant advantage for OTC hearing aids in the dementia caregiving context.

Our participants’ preference for lower-cost, more affordable hearing aids is unsurprising and consistent with the underlying rationale for OTC hearing aids. However, our family caregiver participants made an important distinction between value and cost. To date, most studies on barriers to hearing aid adoption emphasize cost and affordability. Research, however, has shown that value perceptions can also drive hearing aid purchase decisions [57,58]. For older adults broadly, perceived hearing aid value may include emotional, social, quality, and price considerations [58]. Our results suggest that these factors may operate differently in the dementia caregiving context, where family caregivers consider their care recipient’s functional limitations when forming expectations of hearing aid benefits relative to price. By lowering the cost of amplification, OTC hearing aids may play a unique role in improving perceived hearing aid value for people with dementia and their family caregivers.

Our participants’ emphasis on control and patient autonomy is consistent with the priorities of early policy and critique documents that led to the formal introduction of OTC hearing aids, though somewhat less consistent with the OTC hearing aid regulations themselves [59]. Autonomy is seldom discussed in the context of OTC hearing aids, possibly because it is viewed as an aspect of accessibility. Indeed, accessibility does contribute to the ease of obtaining OTC hearing aids. However, our family caregivers and care professionals viewed control as having significance beyond more easily obtaining hearing aids. Specifically, our participants emphasized the importance of autonomy for overcoming stigma-related barriers to hearing aid adoption in people with dementia. Among older adults, dementia and hearing loss each carry stigma—but together, their impact may be even more harmful. For older adults living with both dementia and hearing loss, the 2 stigmas may combine to produce unique fears around loss of independence, aging, and social isolation [60-65]. Although the adoption of OTC hearing aids still requires some acknowledgment of hearing loss, these devices may help overcome stigma by giving older people with dementia and their caregivers the autonomy to circumvent formal medical documentation of hearing loss and hearing aid use.

### Barriers to OTC Hearing Aid Use in People With Dementia

Although participants endorsed several facilitators of OTC hearing aid use in community-dwelling older people with dementia, they also described meaningful barriers that tempered their enthusiasm. From our participants’ responses, we identified the following barriers or disadvantages of OTC hearing aid use: mistrust of OTC hearing aids; assessing OTC hearing aid candidacy in people with dementia; caregiver-facilitated programming/adjustment; assessing the effectiveness of OTC hearing aids; and ongoing OTC hearing aid use and caregiver burden/burnout.

Our findings broadly support and add nuance to the emerging literature on consumer attitudes toward and barriers to OTC hearing aid use. In our study, mistrust emerged as a substantial barrier to OTC hearing aid use for people with dementia and their family caregivers. A recent study of consumer attitudes toward OTC hearing aids also describes trust barriers to OTC hearing aid use. In a survey of 1377 adults without prior hearing aid experience, Singh and Dhar [66] found overall low interest in OTC hearing aids, which they largely attributed to a lack of knowledge and trust in OTC hearing aids. In our study, some participants’ mistrust stemmed from misinformation or lack of knowledge, such as the belief that OTC hearing aids are simple, one-size-fits-all amplifiers. However, participants also conveyed deeper mistrust regarding the competence, motivations, and ethics of those who sell and service OTC hearing aids. Here, it is plausible that people with dementia and their family caregivers may have heightened mistrust of OTC hearing aids compared with the general population. Research indicates that individuals with dementia are particularly vulnerable to financial fraud and exploitation [67,68], potentially leading those with earlier-stage disease and higher self-awareness to approach new products and sales claims with increased caution. Additionally, family dementia caregivers have been shown to harbor mistrust for a variety of dementia care services and interventions, stemming from previous negative experiences and a strong desire to protect their care recipients [69,70].

As discussed previously, both family caregivers and care professionals raised serious concerns about the accuracy and reliability of self-perceived hearing loss in people with dementia. This concern is supported by numerous studies in the general older adult population demonstrating that even without known cognitive impairment, self-reported hearing ability is generally unreliable compared with measured audiometric thresholds [71-73]. However, as our participants highlighted, this problem may be even more pronounced in people with dementia. In a recent study, Kim et al [74] found lower concordance between self-reported and audiometric hearing status in older adults with cognitive impairment than in those without. Notably, the authors also found poor sensitivity of proxy-reported hearing ability in older adults with cognitive impairment, suggesting that family or friends’ hearing loss judgments are an unreliable substitute for self-report in this population [74]. Our qualitative results echo these findings, indicating that family caregivers not only lack confidence in their care recipient’s self-reported hearing status—but also in their own ability to assess and categorize their care recipients’ hearing and communication abilities. Interestingly, our family caregiver participants attributed this lack of confidence, in large part, to the challenge of distinguishing the effects of hearing loss and dementia on communication—an observation echoed in other published studies [31,75]. This insight could help explain the finding of Kim et al [74] of poor sensitivity for proxy-reported hearing loss in their sample of older people with dementia.

Our participants also expressed concerns about the process of programming and adjusting OTC hearing aids. Participants in all 3 groups agreed that people with dementia cannot independently program and adjust OTC hearing aids. This sentiment is well supported by a host of studies documenting the myriad challenges and barriers faced by people with dementia when using both everyday and assistive technologies [35-37]. However, our family caregiver participants also lacked confidence in their own ability to assist their care recipients with OTC hearing aid programming.

While there are few studies of OTC hearing aid use in community-dwelling dementia caregiving dyads, Convery et al [76] found that in a sample of older adults with a range of cognitive abilities, lay partner participation in the OTC hearing aid self-fitting process did not significantly influence or improve outcomes. The authors suggest that fitting outcomes might have improved with knowledgeable support from trained personnel [76]. Here, it is important to consider—as some of our participants mentioned—that many partners and caregivers of older people with co-occurring dementia and hearing loss are themselves older adults who may encounter challenges in using technology. Studies show that older adults face a variety of barriers to technology use, including lack of knowledge, age-related vision loss, and fine motor difficulties, as well as negative attitudes and anxiety toward technology [77-79]. Our family caregiver participants, who were primarily older adults, exemplified these barriers, expressing great apprehension about their technology proficiency and hearing aid knowledge. Notably, in this context, our caregivers’ concerns about technology use were compounded by the previously mentioned barrier surrounding self- and proxy-report of hearing ability in people with dementia. In short, our family caregivers lacked confidence in their care recipients’ ability to self-report changes in hearing to guide caregivers’ programming decisions, and caregivers were also unsure if they could intuit whether they made the correct programming decisions.

Across interest-holder groups, participants were unsure if they could accurately assess the effectiveness of OTC hearing aids in people with dementia. Specifically, our participants raised concerns that self- or caregiver-programmed settings might be suboptimal in ways that could go undetected—potentially leading to sensory agitation, missed benefits, and hearing aid abandonment in people with dementia. To our knowledge, no research has examined the effectiveness of self- or caregiver-fit OTC hearing aids in people with dementia. In the general older adult population, self-programmed OTC hearing aids have consistently shown comparable outcomes to prescription hearing aids [1-5]. However, outcomes of OTC hearing aids in dementia caregiving dyads are yet unknown. This uncertainty may have contributed to our participants’ strong preference for prescription hearing aids and professional guidance. However, this finding is broadly consistent with the results of recent consumer surveys, which show that most adults with self-reported hearing difficulty prefer having professional assistance with their health care [66,80]. Research is urgently needed to determine how OTC hearing aid outcomes compare to those of prescription hearing aids in community-dwelling people with dementia and their family caregivers.

Last, our family caregiver and care professional participants stressed the need for ongoing caregiver involvement to support long-term OTC hearing aid use in people with dementia. Here, our family caregiver participants raised serious concerns about adding OTC hearing aid responsibilities to their already significant caregiving load. Many described feeling overwhelmed and burned out, echoing a large and well-established literature on caregiver burden, burnout, and stress among informal dementia caregivers [81-84]. Furthermore, several of our caregiver participants questioned whether OTC hearing aids would be worth their caregiving effort, especially if the OTC hearing benefit was unapparent and their care recipient was apathetic or uncooperative about using hearing aids.

Considerations of caregiver burden/burnout augment the value theme described earlier to include caregiver perceptions of their work/effort for OTC hearing aid use versus the expected or observed OTC hearing aid benefit. Caregivers may use this calculation of work/effort versus benefit to decide whether to (1) initiate OTC hearing aid use for their care recipient, or (2) continue using OTC hearing aids with their care recipient. Research shows that although people with dementia prefer to be included in important decisions, caregivers often make important medical and treatment decisions for them [85]. In this case, caregivers may ultimately decide whether to invest time and energy into using OTC hearing aids with and for their care recipient. It is important to note that this situation is not unique to OTC hearing aids; it also applies to prescription hearing aids. Indeed, research shows that caregiver assistance is an important determinant of successful prescription hearing aid use in people with dementia [42]. However, the lack of professional support and services for OTC hearing aids could add additional stress on caregivers, potentially increasing the caregiver burden involved in hearing aid use. Research is needed that compares caregiver outcomes for prescription versus OTC hearing aids.

### Clinical Implications and Future Directions

#### Overview

Findings from this study highlight several ways in which OTC hearing aid devices and service delivery models could be better aligned with the needs of people with dementia and their caregivers. Unless and until major cost and accessibility barriers to prescription hearing aids are removed, people with dementia may turn to OTC hearing aids to afford and access hearing health care. Thus, addressing the barriers identified in this study is essential.

#### Implications for Device Design

Participants emphasized perceived challenges related to device complexity, difficult-to-navigate controls, and unclear or insufficient user feedback during use. These findings point to actionable opportunities for OTC hearing aid manufacturers to improve usability for people with dementia and their caregivers. Design considerations informed by participant perspectives include the following:

- Simplified, intuitive programming and troubleshooting workflows that reduce the cognitive load required to set up and adjust devices.
- Dedicated caregiver-assisted programming pathways that allow caregivers to complete or oversee key steps in the setup and adjustment process.
- Caregiver-oriented tools or interfaces that help validate and verify whether amplification settings are appropriate and functioning as intended.
- Clear, interpretable system feedback that communicates both correct and incorrect steps in formats accessible to users with dementia and to caregivers providing assistance.
- Physical design features that minimize handling complexity, including clearly marked controls and components that are easy for both people with dementia and caregivers to understand and manipulate.

#### Implications for Service Delivery and Caregiver Support

Participants consistently emphasized that people with dementia are unlikely to use OTC hearing aids successfully without meaningful caregiver involvement. Perceived challenges included difficulties with initial setup, troubleshooting, and day-to-day management of the devices. These findings point to several opportunities for strengthening service delivery models and caregiver supports:

- Brief caregiver-focused training modules delivered in person, by phone, or through app-based tutorials to assist with device setup, programming, and troubleshooting.
- Simplified written, visual, or video-based instructions tailored to dementia caregiving dyads that reduce cognitive load and clarify step-by-step processes.
- Optional caregiver-tailored remote onboarding or coaching sessions to compensate for the absence of professional fitting services, especially during initial use.
- Caregiver-centered guidance and expectation-setting, including strategies for monitoring benefit, recognizing device malfunction, and supporting consistent use.

Together, these supports reflect opportunities for behavioral and educational interventions aimed at reducing caregiver burden and improving the likelihood of successful device use.

#### Future Research Directions

Further research is needed to evaluate the feasibility and acceptability of OTC hearing aids for people living with dementia and their caregivers. Priority areas include the following:

- Hands-on trials in both laboratory and home settings to observe device use, errors, and points of breakdown.
- Quantifying the magnitude and consequences of identified barriers, including which barriers are most consequential for real-world outcomes.
- Examining caregiver capabilities and roles, including how caregiver burden, health literacy, and technology experience shape outcomes.
- Developing and pilot-testing tailored interventions, device refinements, and service delivery supports informed by the themes identified in this study.

In addition, research is needed to better understand the effectiveness of OTC hearing aids for people living with dementia compared with traditional prescription hearing aids. Clinical trials should evaluate outcomes and benefit domains to clarify when, how, and for whom OTC hearing aids may provide appropriate amplification to support communicative function.

### Limitations

A key strength of this study is its inclusion of multiple different perspectives, providing a more robust exploration of the question than any one interest-holder group alone. However, several important limitations should be noted.

One consideration is that most of our participants with dementia were current hearing aid users. This outcome resulted from our inclusion criterion requiring that participants with dementia self-report mild-to-moderate hearing difficulty. During recruitment, we found that people with dementia who already used hearing aids were more likely to self-identify as having hearing difficulty than those who did not use hearing aids. Additionally, our sample of participants with dementia was predominantly male. This imbalance may reflect the dynamics of our caregiver-mediated recruitment pathways, in which caregivers and community partners more frequently referred male care recipients. Gender differences in the prevalence of age-related hearing loss may also have contributed to this pattern [8].

We also relied on self- or caregiver-reported hearing loss and dementia status rather than confirmed medical diagnoses. This approach was chosen to ensure the feasibility and accessibility of this study for dementia caregivers and people with dementia, for whom additional testing or records requests would have been cumbersome and likely infeasible. In terms of hearing loss, our use of self-reported hearing ability aligns with OTC hearing aid candidacy, which does not require a formal hearing evaluation. Although audiometric results would have been informative for cross-checking self- and caregiver-reported hearing ability, all participants reported mild-to-moderate hearing difficulty consistent with OTC hearing aid candidacy guidelines. Regarding dementia status, our reliance on self- or caregiver reports introduces some uncertainty regarding participants’ cognitive abilities and underlying conditions. However, the combination of self- or caregiver report with the TICS-m cognitive test increases confidence that our sample includes people with early to midstage dementia.

Finally, our study required that participants speak English, limiting the potential racial and ethnic diversity of our sample. Family caregivers and people with dementia who do not speak English may face distinct challenges in using OTC hearing aids, which warrants further investigation.

Given these limitations, caution is warranted when generalizing the findings to women, new hearing aid users, and individuals who do not speak English. Even so, the themes identified in this study point to several opportunities to improve the user experience for many potential OTC hearing aid users with dementia. Future research should more comprehensively characterize the needs of users from different backgrounds and with different levels of hearing aid experience.

### Conclusions

This study identified and described interest-holder-perceived facilitators and barriers to OTC hearing aid use in community-dwelling people with dementia and their family caregivers. Key facilitators or advantages of OTC hearing aid use included improved accessibility and affordability, as well as enhanced autonomy and control. However, significant barriers underscore the complexity of OTC hearing aid use in this population. Interest-holder–perceived barriers included mistrust of OTC hearing aids; assessing OTC hearing aid candidacy in people with dementia; caregiver-facilitated programming/adjustment; assessing the effectiveness of OTC hearing aids; and ongoing OTC hearing aid use and caregiver burden/burnout. Future research should further explore and quantify these and other barriers toward the development of tailored devices, services, and supports that promote successful OTC hearing aid use in people with dementia and their family caregivers. Such work is essential for ensuring that people with dementia can equitably benefit from the introduction of lower-cost, more accessible OTC hearing aids.

## Appendix

Multimedia Appendix 1 Semistructured interview guides.

## Abbreviations

COREQ: Consolidated Criteria for Reporting Qualitative Research
FDA: Food and Drug Administration
HHIE: Hearing Handicap Inventory for the Elderly
IADL: instrumental activities of daily living
OTC: over-the-counter
TICS-m: Telephone Interview for Cognitive Status–modified

## Data Availability

The qualitative data generated and analyzed during this study are not publicly available in order to protect participant confidentiality, as the data cannot be sufficiently deidentified. Researchers interested in further information about the study procedures or analytic approach may contact the corresponding author.
