# Supplementary material for: Facilitators and Barriers to Over-the-Counter Hearing Aid Use in People With Dementia: Semistructured Interview Study
Source: JMIR Hum Factors. 2026 Apr 1;13:e83857. doi: 10.2196/83857 (PMC13085983; doi:10.2196/83857)
Supplement: Multimedia Appendix 1 [file humanfactors_v13i1e83857_app1.docx]

**Appendix: Interview Guides**

**Persons with Dementia**

1. Had you heard about over-the-counter hearing aids?
2. Do you think over-the-counter hearing aids would be useful to you?
3. If you decide to buy hearing aids, how will you decide whether to purchase them from a hearing care professional or purchase them over the counter?
4. If you wanted to buy over-the-counter hearing aids, where would you look for them?
5. What characteristics would you look for in over-the-counter hearing aids?
6. Imagine you purchased over-the-counter hearing aids. They come in a box, and you have the box in your home. You need to take the hearing aids out of the box, turn them on, and put them in your ears. Would you set them up on your own?
   1. *If no*, who would you ask for help?
   2. Are you confident they could help?
   3. What kinds of things would they need to help you set up the hearing aids successfully?
7. Do you think over-the-counter hearing aids would be easy for you to use?
   1. What kinds of things could make them easier to use?
8. Imagine you’ve started wearing over-the-counter hearing aids. Could you tell if they were helping your hearing?
   1. *If yes*, how would you tell?
   2. *If no*, why not?
9. If you purchased over-the-counter hearing aids and weren’t happy with them, what would you do?
10. Imagine you purchased over-the-counter hearing aids and you’ve decided to keep wearing them. What tasks would you need to perform to use the hearing aids successfully?
    1. Which hearing aid tasks could you do on your own?
    2. Which hearing aid tasks do you think you might need help with?
    3. Who would you ask for help?
    4. Are you confident they could help?
11. For you, what are the advantages of over-the-counter hearing aids?
12. For you, what are the disadvantages of over-the-counter hearing aids?
13. Is there anything else about this topic that you’d like to share?

**Family Caregivers**

1. Had you heard about over-the-counter hearing aids?
2. Do you think over-the-counter hearing aids would be useful to [care recipient]?
3. If you and [care recipient] decide to buy hearing aids, how will you decide whether to purchase them from a hearing care professional or purchase them over the counter?
4. Over-the-counter hearing aids are intended for adults with self-perceived mild-to-moderate hearing loss. Do you think [care recipient] can tell their degree of hearing loss?
   1. Can you tell their degree of hearing loss?
   2. Could you help [care recipient] determine their degree of hearing loss? How?
5. If you and [care recipient] wanted to purchase over-the-counter hearing aids, who would primarily shop for them?
   1. *[If primarily the care recipient]*, where do you think they would look for over-the-counter hearing aids?
   2. *[If primarily the family caregiver]*, how would you go about looking for over-the-counter hearing aids? Where would you shop for over-the-counter hearing aids?
6. In the shopping process, what characteristics would you look for in over-the-counter hearing aids that you would consider buying for [care recipient]?
   1. What characteristics of over-the-counter hearing aids would be must-haves for [care recipient]?
   2. Are there any characteristics of over-the-counter hearing aids that would stop you from buying them for [care recipient]?
   3. What do you think [care recipient] would look for in over-the-counter hearing aids?
7. Imagine you purchased over-the-counter hearing aids. They come in a box, and you have the box in your home. The hearing aids need to be unboxed, turned on, and placed in [care recipient’s] ears. Who would be primarily responsible for setting up the hearing aids?
   1. *If primarily care recipient*, what kinds of things would they need to be successful in setting up the devices?
   2. *If primarily family caregiver*, what kinds of things would you need to help [care recipient] set up the hearing aids successfully?
   3. *If primarily family caregiver*, are you confident that you could set up the devices successfully?
8. Do you think over-the-counter hearing aids would be easy for [care recipient] to use?
   1. If you are helping [care recipient], do you think they will be easy for you to use?
   2. What might make over-the-counter hearing aids easier to use?
9. Imagine [care recipient] has started wearing over-the-counter hearing aids. Could you tell if they were helping [care recipient’s] hearing?
   1. *If yes*, how would you tell?
   2. *If no*, why not?
10. Imagine [care recipient] has started wearing over-the-counter hearing aids. Do you think [care recipient] could tell if the hearing aids were helping their hearing?
    1. *If yes*, how would they tell?
    2. *If no*, why not?
11. If you and [care recipient] purchased over-the-counter hearing aids and weren’t happy with them, what would you do?
    1. What do you think [care recipient] would do if they weren’t happy with the devices?
12. Imagine you purchased over-the-counter hearing aids and you and [care recipient] have decided to keep wearing them. What tasks do you think [care recipient] would be able to complete independently to use the hearing aids successfully?
    1. Which hearing aid tasks do you think they might need help with?
    2. Are you confident providing this help?
13. For you and [care recipient], what are the advantages of over-the-counter hearing aids?
14. For you and [care recipient], what are the disadvantages of over-the-counter hearing aids?
15. Is there anything else about this topic that you’d like to share?

**Direct Care Professionals**

1. Had you heard about over-the-counter hearing aids?
2. Do you think over-the-counter hearing aids would be useful to older adults with dementia?
3. If you were providing services to an older adult with early to mid-stage dementia and they or their family caregiver asked your opinion, would you recommend they purchase a hearing aid over the counter or from a licensed hearing care provider?
4. Over-the-counter hearing aids are intended for adults with mild-to-moderate hearing loss. Imagine an individual with early to mid-stage dementia and/or their family caregiver want to determine if the care recipient’s hearing is appropriate for over-the-counter hearing aids. Do you think the individual with dementia could assess their degree of hearing loss?
   1. Could a family caregiver determine the individual’s degree of hearing loss?
   2. Do you feel confident classifying the hearing status of individuals under your care?
   3. How would you make this determination?
5. Imagine that the care recipient and their family caregiver decide to purchase over-the-counter hearing aids. They ask you where to find over-the-counter hearing aids. How would you recommend they go about looking for over-the-counter hearing aids?
6. For individuals under your care with early to mid-stage dementia, what would you recommend they or their family caregivers look for in over-the-counter hearing aids?
   1. What features or characteristics might they need to be successful with the devices?
   2. Are there features or characteristics you think could be detrimental for these individuals?
   3. Do you think individuals with dementia will be self-aware of these factors?
   4. How about their family caregivers?
7. Imagine the care recipient and/or their family caregiver purchased over-the-counter hearing aids. They now have the box in their home. Do you think the person with dementia could independently set up the hearing aids? In other words, take the hearing aids out of the box, turn them on, place them on their ears, and start wearing them.
   1. Do you think a family caregiver could assist them in setting up the hearing aids?
   2. What kinds of things do you think the person setting up the hearing aids would need to be successful?
8. Do you think over-the-counter hearing aids will be easy to use for individuals with early to mid-stage dementia?
   1. What things could make them easier to use for this population?
9. Imagine a person under your care with early to mid-stage dementia has started wearing over-the-counter hearing aids. Could you tell if the hearing aids were helping the person hear better?
   1. How would you tell?
   2. Do you think a family caregiver could tell that the hearing aids were helping? And how?
10. Thinking of people under your care with early to mid-stage dementia, do you think they could tell if over-the-counter hearing aids were helping their hearing?
    1. *If yes*, how would they tell?
    2. *If no*, why not?
11. Imagine the care recipient and their family caregiver decide they aren’t satisfied with the over-the-counter hearing aids they purchased; what would you recommend they do?
12. Thinking of individuals under your care with early to mid-stage dementia, do you think they could handle and maintain over-the-counter hearing aids independently?
    1. Which hearing aid tasks do you think they might need help with?
    2. Do you think family caregivers could help with these tasks?
    3. Could you help these tasks?
13. From your perspective as a care professional, what are the advantages of over-the-counter hearing aids for individuals with early to mid-stage dementia?
14. From your perspective as a care professional, what are the disadvantages of over-the-counter hearing aids for individuals with early to mid-stage dementia?
15. Is there anything else about this topic that you’d like to share?
